# P-1843. Genomic surveillance of invasive *Streptococcus pyogenes* in Metropolitan Detroit

**DOI:** 10.1093/ofid/ofae631.2004

**Published:** 2025-01-29

**Authors:** Jagjeet Kaur, Megha Jagannathan, Tamara Jordan, Daniel Kinsey, Maryssa Truppiano, Anita Shallal, Geehan Suleyman

**Affiliations:** Henry Ford Health, Detroit, Michigan; Henry Ford Hospital, Detroit, Michigan; Henry Ford Hospital, Detroit, Michigan; Henry Ford Hospital, Detroit, Michigan; Henry Ford health System, Detroit, Michigan; Henry Ford Health, Detroit, Michigan; Henry Ford Health, Detroit, Michigan

## Abstract

**Background:**

Group A streptococcal (GAS) disease is a major problem worldwide and can cause both noninvasive and invasive disease, including necrotizing fasciitis, with significant morbidity and mortality. Overall, the number of invasive GAS infections has been increasing in the United States over the past decade, primarily in adults. We aim to characterize the genomic features of circulating GAS strains causing invasive disease.

**Table.**
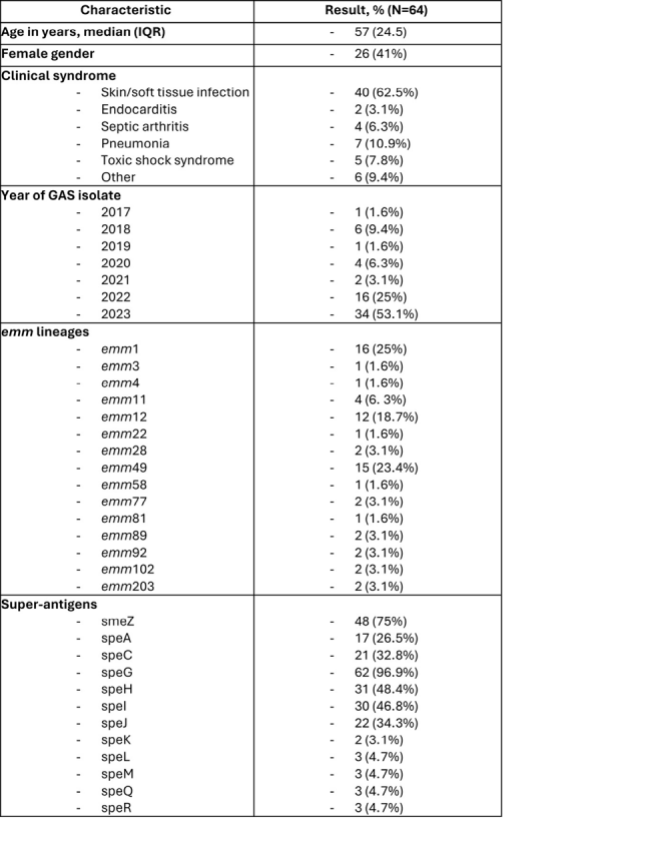
Patient demographics and genomic characteristics

**Methods:**

We performed whole-genome sequencing (WGS) on clinically obtained GAS blood isolates at Henry Ford Health, a comprehensive, integrated, health care organization in Southeast Michigan from Jan 2017-Dec 2023. Sequencing libraries were created using the QIAseq FX DNA Library Kit (Qiagen,USA) according to manufacturer’s instructions and sequenced on NextSeq 2000 (Illumina Sandiego, USA). Fastq files were used to determine the distribution of traits, virulence factors, and resistance genes using the 1928 Platform.

**Results:**

We sequenced unique isolates from 64 patients, of whom 38 (59%) were males with median age 57 years [Table]. Most isolates were from 2022 (25%) and 2023 (53%). Fifteen M protein (emm) serotypes and 19 sequence type (ST) were obtained from 64 characterized isolates. The most frequent emm lineages were emm1 (n=16) and emm12 (n=12), where most patients had skin and soft tissue infection. The predominant emm1 lineage belonged to ST28. None of the emm1 and emm12 isolates carried aminoglycoside, macrolides/lincosamides/streptogramines (MLS), or tetracycline resistance genes. All the emm11 and emm49 isolates exhibited erm(A) and tetracycline resistance genes. Streptococcal superantigen (SAgs) genes were frequently detected; streptococcal pyrogenic exotoxin (SPE)-G was the most detected SAgs (97%), followed by streptococcal mitogenic exotoxin (SME)-Z (75%). SPE-H and SPE-I were each found in 48% of the isolates.

**Conclusion:**

In our cohort of patients with invasive GAS, several SAgs were detected. SPE-A, SPE-G, SME-Z and SPE-J were most frequently found in emm1 lineages. However, there was no correlation between various clinical syndromes and the virulence genes identified. Continued genomic surveillance can help characterize features associated with emerging invasive strains to inform management and infection prevention strategies.

**Disclosures:**

**All Authors**: No reported disclosures

